# Use of different genetic predisposition score techniques to predict muscle mass and muscle function over the adult life span in Flemish Caucasians

**DOI:** 10.1186/2049-3258-73-S1-P37

**Published:** 2015-09-17

**Authors:** Ruben Charlier, Maarten Caspers, Sara Knaeps, Evelien Mertens, Diether Lambrechts, Johan Lefevre, Martine Thomis

**Affiliations:** 1 KU Leuven, Leuven, Belgium; 2 VUB, Brussels, Belgium

## 

Since the Belgian elderly population is expanding at a very high rate, it is important for public health care systems to better understand ageing-related syndromes and diseases, such as sarcopenia. This condition is characterized by a progressive loss of skeletal muscle mass and its action generating capacity (also termed muscle function). Therefore it also implies risk of adverse outcomes like fall-related injury and impaired quality of life. Furthermore, it is well known that both muscle mass and muscle function are under strong genetic determination. However, most previous studies only considered individual associations of one specific gene variant with muscular phenotypes, whereas it is likely several related polymorphisms that combine to influence muscular characteristics. The data of the current study were assessed within the framework of the first (2002-2004) and third (2012-2015) generation Flemish Policy Research Centre Sport. A total of 579 healthy, Flemish Caucasian adults (19-73, 64.4% men) were measured during a follow-up period of ten years. Genomic DNA was extracted and 384 gene variants were genotyped. Force and velocity characteristics of the knee extensors were measured using the Biodex Medical System 3® dynamometer. Furthermore, four genetic predisposition score (GPS) models were designed: a total GPS (based on the sum of all variants, each favorable allele = score 1), a data-driven GPS (sum of favorable alleles of those variants with significant b-coefficients in stepwise regression), a weighted GPS (weighting is done based on the proportion of the variance explained by those variants with significant b-coefficients in stepwise regression) and an elastic-net GPS (sum of favorable alleles included by elastic-net regularization). Afterwards, these GPS models will be compared and used to predict genetic predisposition towards muscle mass and muscle function and its loss by ageing via linear regression analysis.

